# Does oral contraceptive pill increase the risk of abnormal Pap smear?

**Published:** 2013-09

**Authors:** Fariba Binesh, Ali Akhavan, Azar Pirdehghan, Mahnoosh Davoodi

**Affiliations:** 1 *Department of Pathology, Shahid Sadoughi University of Medical Sciences, Yazd, Iran.*; 2 *Department of Radiotherapy, Shahid Sadoughi University of Medical Sciences, Yazd, Iran.*; 3 *Department of Community Medicine, Shahid Sadoughi University of Medical Sciences, Yazd, Iran.*; 4 *Shahid Sadoughi University of Medical Sciences, Yazd, Iran.*

**Keywords:** Oral contraceptive pill (OCP), Pap smear, Abnormal.

## Abstract

**Background:** It is noted that oral contraceptive pills increase the risk of abnormal Pap smear but results have been inconsistent across the populations.

**Objective: **This study aimed to evaluate the association between oral contraceptive pill (OCP) consumption and abnormal Pap smear in women who referred to Shahid Sadoughi and Madar hospitals in Yazd.

**Materials and Methods:** A cross sectional descriptive study was carried out and a database of all Pap smear reports from 2009-2011 at Cytopathology Department of Shahid Sadoughi and Madar hospitals in Yazd, Iran was reviewed. A total number of 1286 women with history of OCP consumption were selected as the case group and 1218 women applying other contraceptive methods were selected as control group for evaluation. Both case and control groups were matched by age, parity and socioeconomic status. All of the women in this study maintained a single partner as their husband and none of them were considered as smokers. The duration of OCP use was at least 5 years.

**Results: **Abnormal Pap smear results were observed in 0.4% of cases and 0.2% of controls. There was no significant association between OCP consumption and abnormal Pap smear (p=0.727).

**Conclusion:** Our findings did not show any specific association between OCP consumption and abnormal Pap smear results. In addition, the number of abnormal Pap smears in women who consumed OCP was lower than that of western countries. More prospective studies are required.

## Introduction

Invasive squamous cell carcinoma of the cervix is still the most common malignant tumor of the female genital tract in most countries (1). Due to the lack of a population-based universal national cancer registry center in Iran, there is no accurate data on the incidence of cancers. However, the prevalence of cervical cancer in Iran seems to be much lower than in other developing countries (2). 

According to WHO declaration cervical cancer ranks as 12th most frequent cancer among women in Iran (3). Cervical dysplasia is a premalignant lesion that can progress to cervical cancer. Invasive cancer of the cervix has been considered a preventable cancer because it has a long pre invasive state, cervical cytology screening programs are available, and the treatment of pre invasive lesions is effective (4). Cytologic screening of cervical smears is the most effective screening test for cervical cancer ever developed (5). 

Over the past several years, through improvements in screening and treatment, the incidence and mortality rates of cervical cancer have dramatically declined in developed countries (6). In spite of the preventable nature of this disease, 12,710 new cases of invasive cervical cancer resulting in 4290 deaths were anticipated in the United States in 2011 (7). Despite new modalities of treatment, up to 35% of patients diagnosed with cervical cancer will subsequently develop metastasis (8). 

An association of cervical cancer with several risk factors including human papilloma virus infection, smoking, oral contraceptive consumption and male factor (multiple partners) have been shown in some studies (9-11). Additionally, it has been propounded that there are some host factors which are effective in progression of disease (12). Oral contraceptive pill (OCP) is the most universal modern method of contraception, followed by female sterilization, in the world (13). 

In Iran, the use of OCP has been increased specially in the last two decades and is available to married couples, free of charge, at public clinics (14). Over the last 40 years, literature regarding OCPs has accentuated the “dark side” of the drug and pointed out how their numerous side effects adversely impact many users and even societies at large. A large body of epidemiological evidence suggests that cervical cancer risk might increase by using OCPs for a long period (15-17).

But results have been inconsistent across populations. For example in some studies (some of them are from Iran) no consistent association emerged between the risk of intraepithelial cervical neoplasm and oral contraceptive use (18-20). On the other hand there are some studies which suggest that OCP consuming for a long time, might raise cervical cancer risk (21-24). In this study, we investigated the effects of combined oral contraceptives on the prevalence of abnormal Pap smear among women who were referred to Shahid Sadoughi and Madar hospitals in Yazd.

## Materials and methods

A cross-sectional descriptive study was conducted over a 2 years period from 2009 to 2011. The study was approved by the university ethics committee. As has been done previously by McFarlane-Anderson *et al* all cytological reports and slides at Cytopathology Department of Shahid Sadoughi and Madar hospitals in Yazd , within this period were reviewed (17). This covered 1286 women who have taken OCP and constituted the case group. The control group included women who had regular Pap test checkups as part of their routine screening during the same period of the study and they had not used OCP. 

Both the case group and the control group were recruited from the same community and were demographically similar. Cervical samples from the subjects had been taken after oral consent. Women who were previously diagnosed with cervical cancer or who with known premalignant lesions and who have been received injectable contraceptive compound were excluded. The age of the cases and of the controls was matched. The age range for both groups was limited between 25 and 60 years. The duration of using the pills was at least 5 years. Abnormal Pap tests included atypical squamous cells of undetermined significance (ASCUS), dysplasia and carcinoma. 

The 2001 Bethesda system was used to classify the epithelial abnormalities (25). Equipment indispensable for HPV DNA testing was not available in our laboratory and therefore, the procedure was not performed. Clinical data with regard to smoking, duration of OCP use and marital status, when available and accessible, were collected for the case and control. Since our local community is a rather conservative, we assumed that all our women do not smoke, do not drink alcohol and are not sexually promiscuous.

**Statistical analysis**

Statistical analysis included Chi-Squared test and Fischer’s exact test. The odds ratio (OR) and 95% confidence interval (95% CI) were applied to estimate the relative risk of negative and positive Pap tests of the cases and the controls. Test results with a probability p<0.05 were considered to be statistically significant. Statistical Package for Social Sciences (SPSS, Chicago, IL) version 15.0 software was used to perform the statistical analysis.

## Results

There were 1286 cases and 1218 controls involved in this study. Ages ranged from 25-60 years with a mean age of 35 years. Demographic characteristics are depicted (Table I). The mean ages of the cases and the controls were 34.14±6.48 and 35.93±7.56, respectively. 

Five (0.4%) women who had used OCP showed abnormal Pap tests. All of these abnormal Pap tests indicated ASCUS. The control group had three (0.2%) women with abnormal Pap tests in which two of the cytology reports were compatible with ASCUS and one result disclosed low- grade squamous intraepithelial lesion (LSIL). According to the calculated p-value (p=0.727), no association between OCP consumption and abnormal Pap smear was observed. 

The estimated Odds ratio for the cases (OR=1.579, 95% CI=0.378-6.591) was not significantly superior to the estimated Odds ratio for the controls (OR=0.999, 95% CI=0.996-1.003). The mean duration of OCP consumption in women with normal Pap smear was 7.66±2.6 years and in women with abnormal Pap smear was 10.8±3.6 years. According to p=0.019 there is a significant difference between women with normal and abnormal Pap smear regarding duration of OCP consumption.

**Table I:** Patients characteristics and Pap smear results in both groups

		**Method of Pregnancy prevention**		**p-value**
		**OCP (n=1286)**	**Non-ocp (n=1218)**	
Age mean± SD		34.14 ± 6.48	35.93 ± 7.56	<0.001
Pap smear				0.727
	Positive	5 (0.40%)	3 (0.20%)	
	Negative	1281 (99.60%)	1215 (99.80%)	

## Discussion

In this study we found no association between OCP consumption and abnormal Pap smear. The possible association between the use of OCP and the development of cervical neoplasia has been the subject of many epidemiological investigations, but the nature of the association remains unclear (26). A remarkable amount of epidemiologic evidence suggests that OCP consuming for a long time, might raise cervical cancer risk, but results have been inconsistent across populations (21). 

In addition, it is difficult to separate the effect of oral contraceptive pill use on the risk of contracting HPV infection. Animal studies has shown that Rhesus monkeys that were given high doses of medroxy progesterone developed cervical cancer (27). Furthermore, in 1993 a massive worldwide study conducted by the World Health Organization was published which examined the risk established between OCP use and invasive squamous cervical carcinoma among 2,300 women who had cervical carcinoma and found a strong correlation (22). 

In a large study group, Herrero *et al* showed that women who had received injectable progestins for at least 5 years and who had used them at least 5 years ago suffered a 430% increased risk of developing cervical cancer (23). Also, Briton revealed that usage of OCP for more than 10 years could increase the risk of cervical cancer (24). One study has shown that current use of combined OCPs, a positive HIV test and multiparity are significant predictors of high-grade cervical lesions (11). 

On the other hand, in some studies this association has fallen in to suspicion. Molina and coworkers did not find any risk for neoplasia in women who ever used OCP after controlling for the possible confounders and two other studies even showed a protective effect of OCP on severe cervical dysplasia (18-20). The inconsistent reports of an association between hormonal contraception and cervical dysplasia and cancer may be related, in part, to confounding risk factors that include sexual and lifestyle behaviors (28). These factors are difficult to control.

It should be noted that the causal link between HPV and cervical dysplasia and cancer is now generally accepted (29). The literature is overwhelmed with evidential data supporting that human papilloma virus (HPV) is a necessary etiological factor in the pathogenesis of cervical dysplasia and carcinoma (30). However, it is believed that a high proportion of HPV-infected women do not develop serious cervical neoplasia and that other co-factors may be necessary to ultimately culminate severe disease (31). OCP use has been suggested as one possible factor (32). 

Other cofactors that may play a role in the carcinogenesis process include smoking, other sexually transmitted diseases and prolonged immunosuppression (33). Given that HPV infection is now considered the major factor in development of cervical cancer, it would seem that OCP may be acting as an enhancer of neoplastic growth. In addition to any direct effect of OCP on the development of cervical dysplasia, either as an initiator or promoter of carcinogenesis, the use of hormonal contraceptives could result in women indulging in more unprotected sexual activity, putting them at more risk of HPV infection and other sexually transmitted infections and their sequel (34). In this study we found no association between OCP consumption and abnormal Pap smear. 

To explain these results, it should be taken into account that pathogenesis of cervical cancer and precancerous lesions in Muslim countries might be different compared with western societies due to the difference in the risk factors. The incidence of abnormal Pap smear in Iran is relatively low compared with other studies performed in industrialized countries (35). We are in agreement with others, that we do have a relatively lower prevalence of cervical carcinoma and cervical premalignant lesions. In one study the rate of abnormal Pap smear in Iran has been reported to be 0.2-1% (36). This is most likely related to the sexual behaviors conducted under Islamic rules. In Iran, sexual activity typically starts only after marriage and the cultural and religious traditions of our conservative society restrict the likelihood of multiple sexual partners. 

This fact by itself decreases the possibility of HPV and other sexually transmitted infections. However other practices such as male circumcision, which is customary in our country and very low rate of smoking among Iranian women due to cultural context, may play an important role as well. From above-mentioned findings, it is clear that our society has a lower prevalence of HPV infection and cervical neoplasia and as a result, the association between OCP and abnormal Pap smear is weak. On the contrary, in this study there was a significant difference between women with normal and abnormal Pap smear regarding duration of OCP consumption. Thus, long term exposure to OCP conferred more risk for induction of cervical premalignant and malignant changes. 

Moreover, other studies have shown that prolonged use of OCP (more than 8-10 years) had a significant influence on the increased risk of cervical cancer (37). Therefore, women using OCP for a prolonged period of time should be encouraged to do regular Pap smear screening. It should be noted that our study had some limitations: 1) The most important limitation was the lack of information about HPV infection, 2) We used conventional pap smear test which had high rate of false-negative results. 3) There were some difficulties in interview because questions related to sexual and reproductive behaviour are considered taboo in Muslim countries such as Iran.

## Conclusion

Results from current study did not show an association between OCP consumption and abnormal Pap smear results. More prospective studies are required.
